# The unusual association of inverse retinitis pigmentosa and Fuchs’ heterochromic iridocyclitis

**DOI:** 10.1186/s40942-016-0056-5

**Published:** 2017-01-23

**Authors:** Gian Franco Díez-Cattini, David Arturo Ancona-Lezama, Carlos Valdés-Lara, Virgilio Morales-Cantón

**Affiliations:** Servicio de Retina y Vítreo, Hospital Luis Sánchez Bulnes, Asociación para Evitar la Ceguera en México, I.A.P, Vicente García Torres 46, Colonia Barrio San Lucas, 04030 Coyoacan, Mexico City, Mexico

**Keywords:** Retinitis pigmentosa, Fuchs’ heterochromic iridocyclitis, Inverse retinitis pigmentosa, Uveitis

## Abstract

**Background:**

Classic retinitis pigmentosa (RP) and other syndromic variants have previously been associated to Fuchs’ heterochromic iridocyclitis (FHI). Common immunogenic and inflammatory pathways have been proposed to explain the higher incidence of this uveitic phenomenon in patients with retinal dystrophies without definitive answers. Infrequent variants of RP such as inverse RP have not been previously reported in association with FHI. We believe that finding the way these entities connect can shed some light into their complex pathogenesis and help find ways to foresee and prevent the appearance of complications such as cataract and macular edema.

**Case presentation:**

We present a 15 year old mexican male with history of nyctalopia and rapid, progressive visual loss since infancy who had profound hyper and hypopigmented retinal pigment epithelium changes in the posterior pole together with pigment clumping in the macula of both eyes and an electroretinogram pattern consistent of rod-cone dystrophy. He was diagnosed with inverse RP. Three years after his first visit he was found to have a mild asymptomatic non granulomatous anterior uveitis in the right eye with fine stellate keratic precipitates and subtle iris stromal atrophy not associated with iris synechiae and without evidence of posterior uveitis or findings consistent with infectious etiology. Findings were consistent with FHI. As the patient was normotensive, the lens was transparent and there was no clinical evidence of macular edema, the patient was kept under observation without treatment.

**Conclusions:**

Patients with RP are prone to develop chronic, low grade inflammation responses similar to the ones present in FHI. This association makes us believe that immunogenetic pathways involved in the degenerative process that leads to photoreceptor loss may become a target in the prevention and treatment of inflammatory complications in RP and disease progression. It also suggests FHI may be a triggered response predisposed by an unidentified genetic factor that may be related to genes affected in RP and thus be identified before irreversible complications such as glaucoma occur.

## Background

Retinitis pigmentosa (RP) is one of the most common genetic retinal disorders, with an estimated prevalence of 1 in 4000 [1]. It is an heterogeneous dystrophy affecting both rods and cones which is transmitted in either autosomal dominant, autosomal recessive and X-linked inheritance.

Inverse RP, also called RP centralis is a rare form of RP initially affecting the photoreceptors located in the macula, causing important visual impairment in very early stages of presentation. An autosomal recessive inheritance has been postulated but not proven [2]. Many authors concur in the possibility that this rare form of RP may correspond to a cone-rod dystrophy (CRD) with macular hyperpigmentation [2, 3]. Diagnosis is difficult and other inherited retinal disorders such as Leber’s congenital amaurosis, progressive CRD and central areolar choroidal sclerosis should be ruled out.

Fuch’s Heterochromic Iridocyclitis (FHI) is a chronic, idiopathic, and mostly unilateral uveitis that affects both men and women. It is characterized by anterior chamber cell reaction atrophy of the iris stroma. In patients with light colored irides the affected eye turns lighter in color; in dark-colored irides stromal atrophy is more subtle, with flatness of iris crypts, a velvety appearance of the stroma or inverse heterochromia, in which the affected eye turns darker [5]. The anterior uveitis of FHI can present with Koeppe or Busacca nodules but it does not cause synechiae. It is usually accompanied by posterior subcapsular cataract and may be complicated with ocular hypertension in 30% of all cases [5].

The occurrence of RP and FHI in the same patient was first reported by Vourre et al. [6]. Roughly a dozen more case reports have described patients with both coexisting diseases. Chowers et al. [7] published a controlled cohort study comparing RP patients with normal subjects finding an overall difference in the frequency of FHI in both groups (1.2 vs. 5%) definitely proving there was a true association between them and not a coincidental phenomenon.

Since its first description, attempts to link the pathogenesis of both diseases have been made through the observation of some shared clinical characteristics such as the presence of posterior subcapsular cataracts and macular edema. It has been found that cellular and humoral immune responses towards retinal antigens in patients with RP provoke a mild and chronic inflammatory reaction thought to be responsible for some of its clinical findings: namely macular edema and vascular leakage [1]. Other studies have demonstrated the presence of B-cells against corneal, retinal and anterior segment antigens in the aqueous of patients with FHI [4, 5].

FHI etiology remains unknown, and even though several mechanisms have been proposed as triggers of the characteristic inflammatory reaction [4], all of them have failed to explain the tendency to be unilateral in 90% of all cases. This fact gains importance in the light of the association with RP, which is bilateral in almost 100% of the patients.

Knowing both diseases are the result of inflammatory reactions or are accompanied by a tendency to produce low grade chronic inflammation maked us believe that the key common pathogenic clue lies in an unidentified autoimmune, genetic or combined factor.

Usher’s disease, a syndromic form of RP accompanied by congenital deafness, has recently been associated with FHI with a higher incidence than classic RP [8]. Whether the same unknown mechanisms that predispose RP patients to develop FIH are enhanced in Usher syndrome, or there are other contributing factors is unknown to this date. To our knowledge, there are no reports of inverse RP associated with FIH.

## Case presentation

**Fig. 1:**
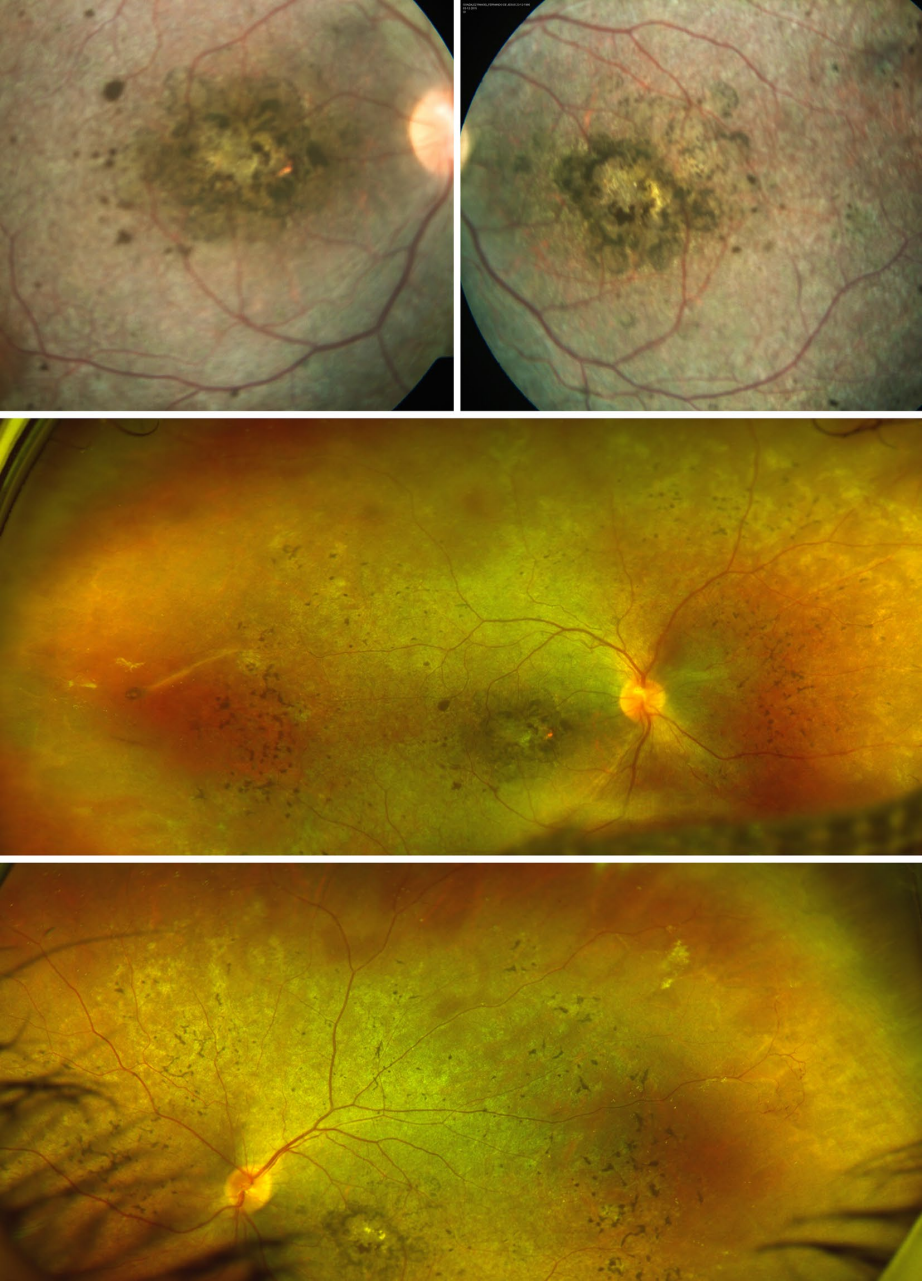
Fundus photographs. Retinal hyperpigmentation in bone spicules at mid-periphery. Pigment clumping in macular area. Extensive atrophy of retinal pigment epithelium. Slight retinal arteriolar attenuation

A 15-year-old male with history of chronic, progressive and bilateral visual loss accompanied by nyctalopia, first noticed at age 6 presented for ophthalmologic evaluation. He had been previously diagnosed with retinal dystrophy. Upon examination, his best corrected visual acuity (BCVA) was counting fingers at 1 m in both eyes. Intraocular pressure was 11 mmHg in the right eye (OD) and 13 mmHg in the left eye (OS). Ocular motility was normal. Anterior segment examination was unremarkable and the lens was transparent. Fundus examination revealed fine pigmented dust in the vitreous gel, hyper and hypopigmented retinal pigment epithelium (RPE) changes forming ‘bone spicules’ scattered in the posterior pole up to the equator and pigment clumping in both macular zones. Arteries and veins were slightly narrow or attenuated (Fig. 1).

**Fig. 2:**
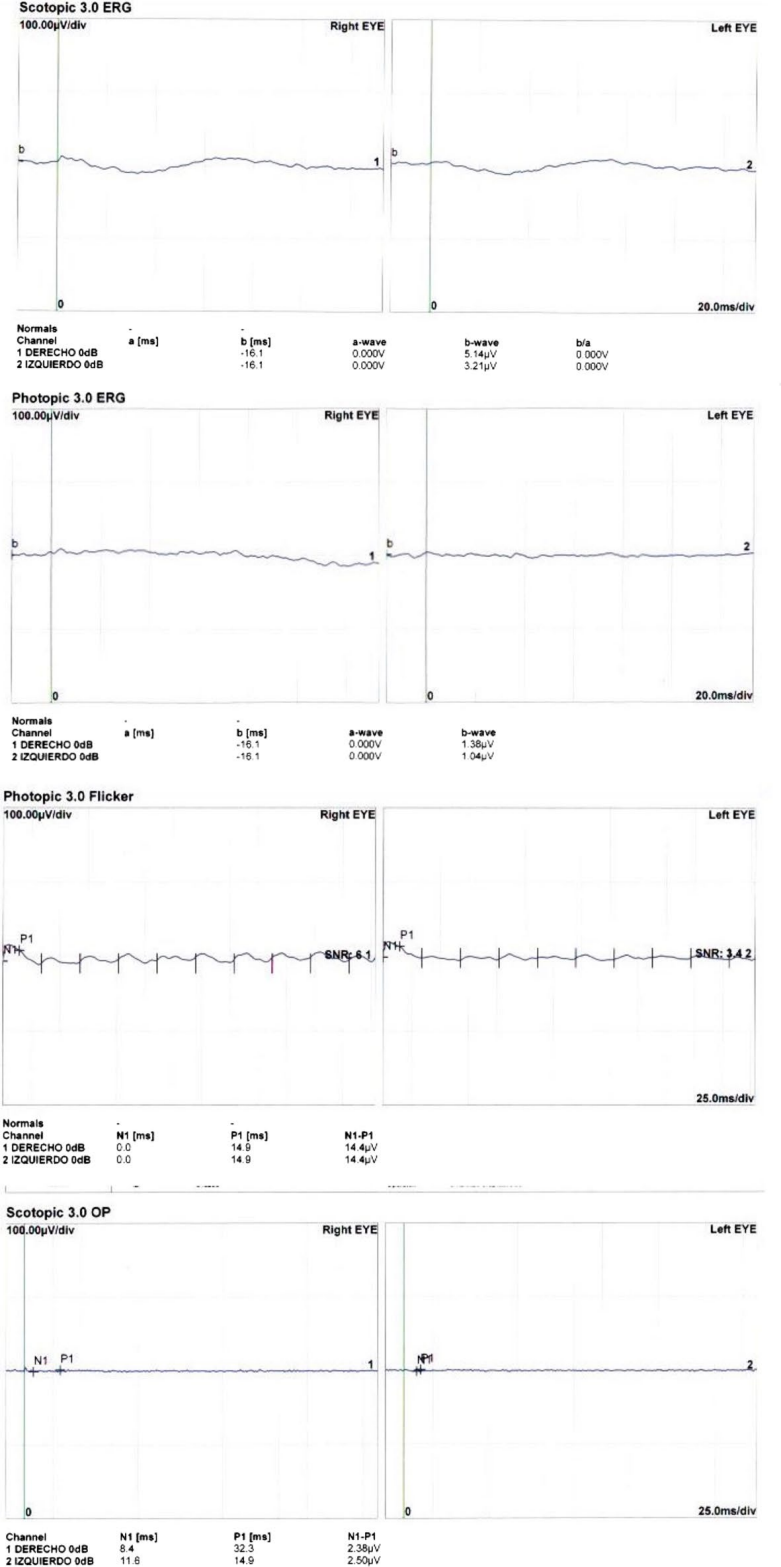
Electrorretinogram. Scotopic and photopic responses with marked reduction in the amplitude of *a* and *b* waves. Flicker and oscillatory potentials with implicit time delay and flattening of recordings

**Fig. 3:**
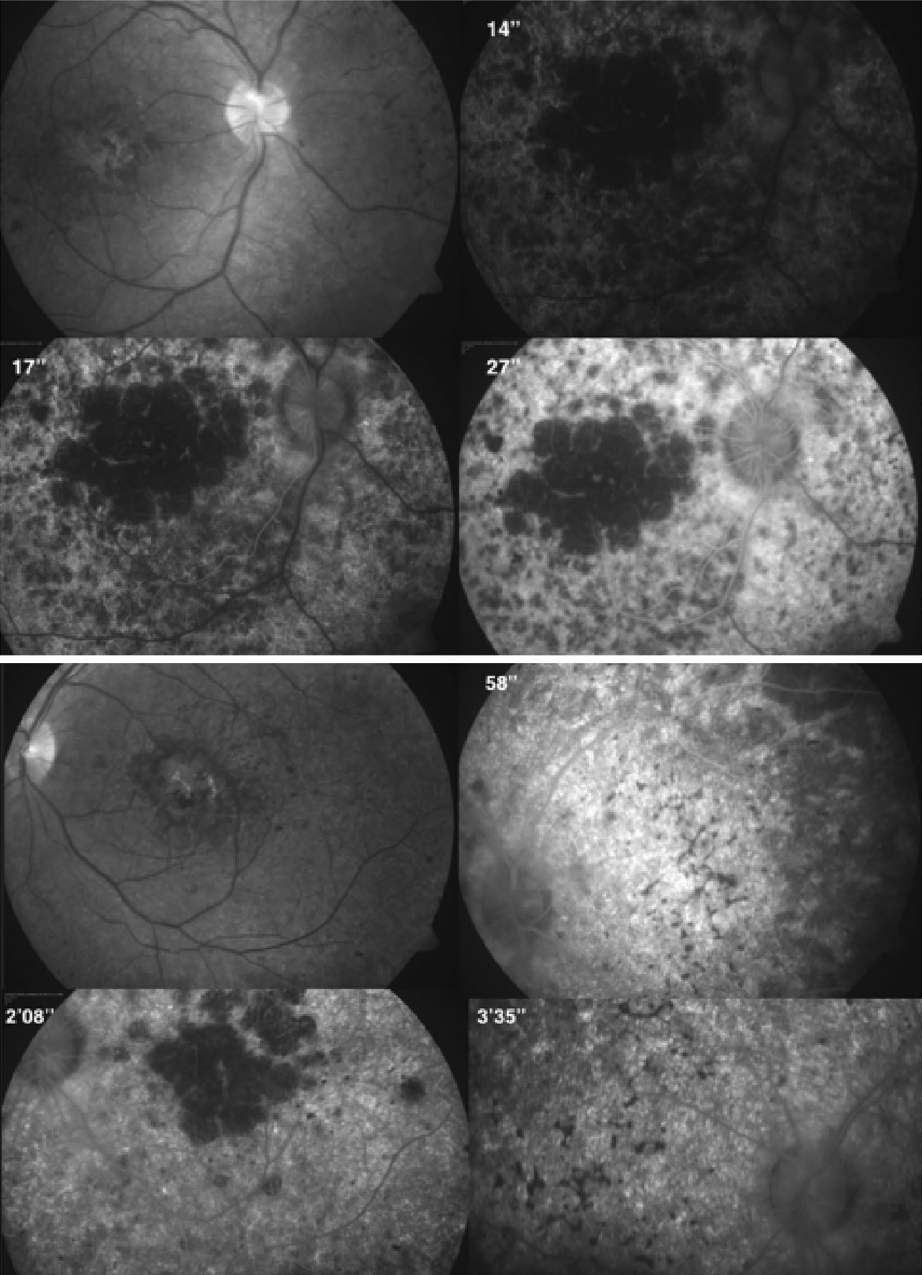
Fluorescein angiography. Macular fluorescence blockage from pigment clumping, patchy areas of hyperfluorescence corresponding to areas of RPE atrophy causing window defect

Electroretinogram (ERG) showed diminished b-wave amplitudes in both photopic and scotopic phases with a marked reduction in the amplitude of all waves, implicit time delay of the flicker response and flattening of oscillatory potentials (Fig. 2). The electrooculogram showed an abnormal RPE response. Automated visual fields registered a generalised decrease in sensitivity and profound central scotomas. Fluorescein angiography evidenced central hypofluorescence due to contrast blockage in the areas with pigment accumulation and patchy hyperfluorescence due to window defects in RPE atrophy areas (Fig. 3).

The patient denied using any medication and had no relevant medical history for infectious or systemic diseases. Fundus changes and abnormal scotopic and photopic ERG responses made the diagnosis particularly difficult. The absence of nystagmus, strabismus or photophobia along with an older age of presentation and slower rate of progression made a diagnosis of Leber’s congenital amaurosis less likely, progressive CRD can have a very similar clinical presentation with ERG photopic responses initially more affected than scotopic traces, a difference that becomes more subtle as the disease progresses until it becomes negligible.

Severe affection of both rods and cones in ERG made a diagnostic distinction difficult. The presence of nyctalopia, no history of photophobia and characteristic appearance of bone spicules oriented the diagnosis towards a case of inverse RP, although CRD with macular hyperpigmentation could not be discarded completely.

The patient started follow up in the retina clinic and was instructed on the use of visual aids and genetic counseling was provided. Regular consultations were scheduled every 6 months without worsening of symptoms or BCVA; all examinations were reported as stable.

**Fig. 4:**
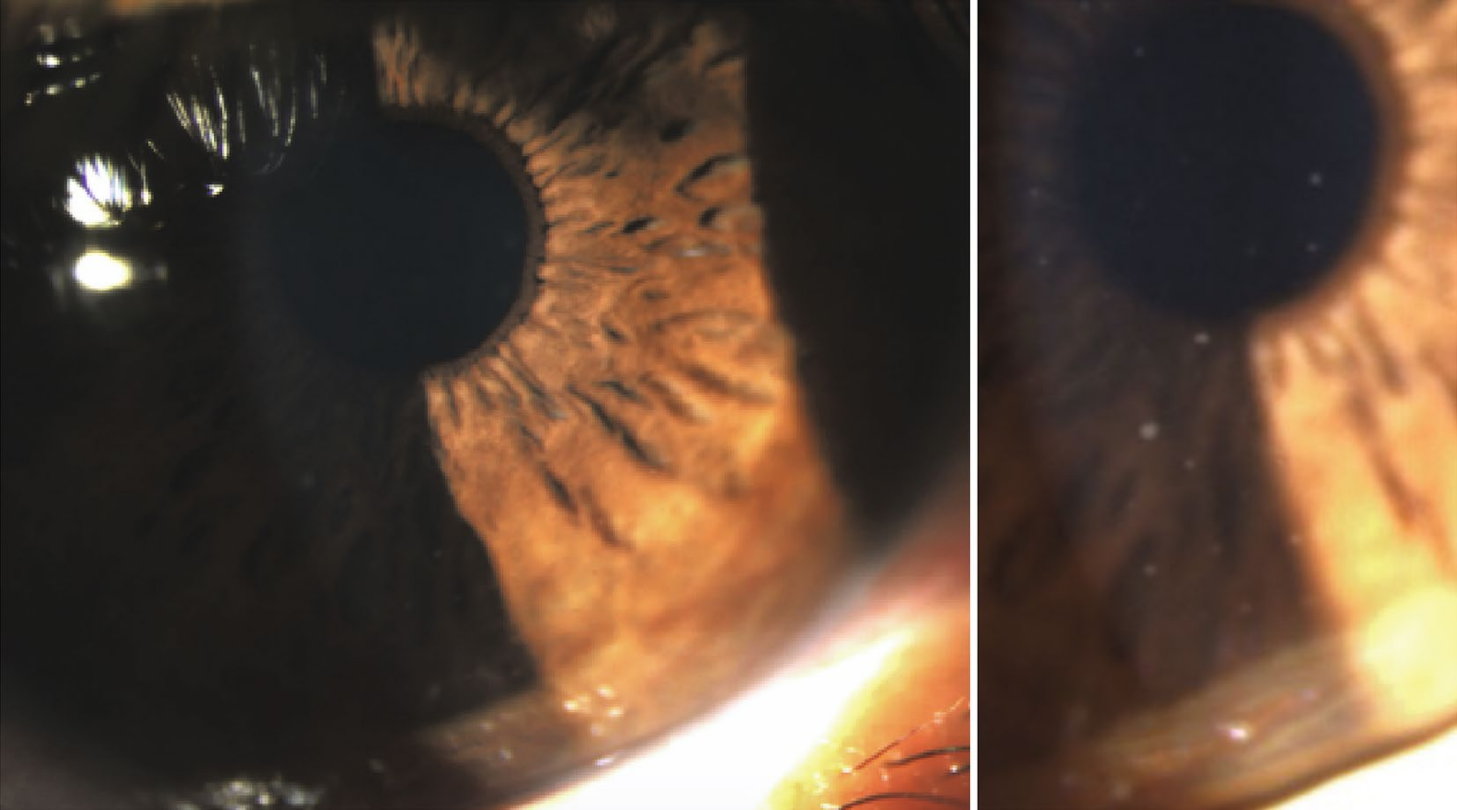
Clinical photograph right eye. Fine diffuse keratic precipitates more evident in the lower cornea. The patient also had mild anterior chamber cellular reaction (0.5+) and a subtle stromal velvety appearance

**Fig. 5:**
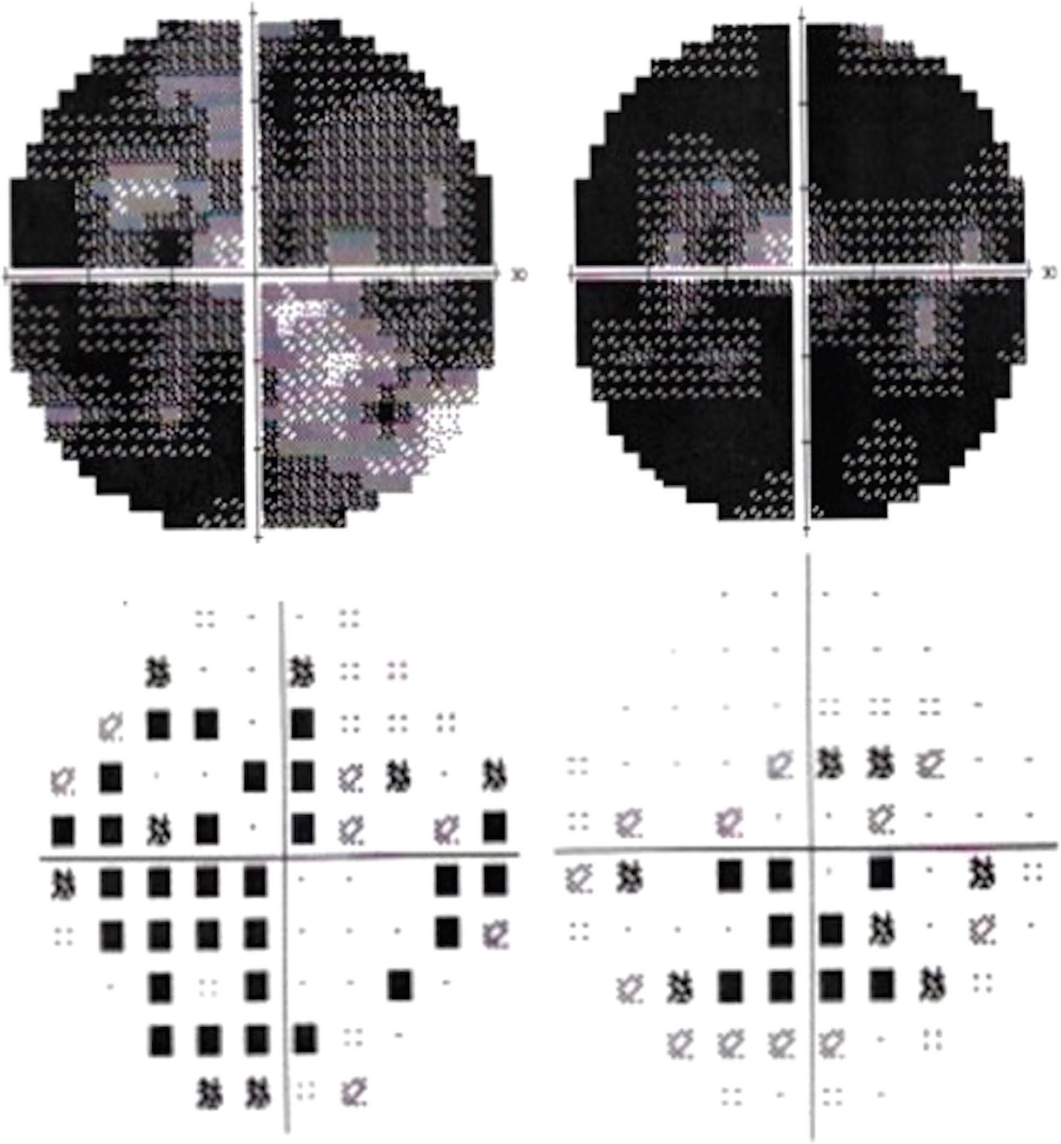
Automated visual fields. Severe affection of both visual fields involving fixation

On a follow-up visit, 3 years after initial consultation, examination of the OD revealed a white asymptomatic eye with fine translucent ‘stellate’ keratic precipitates scattered over the entire endothelium and mild cellular reaction in the anterior chamber with no synechiae, iris nodules or heterochromia but subtle stromal atrophy of the iris crypts in comparison with the OS (Fig. 4). BCVA was unchanged and IOP was 16 mmHg OD and 12 mmHg OS. Dilated fundus exam did not show any sign of vasculitis, retinitis or choroiditis. The OS showed no signs of inflammation thus matching the criteria for unilateral Fuchs’ heterochromic iridocyclitis (FHI) Visual Fields were severely affected (Fig. 5) .

## Conclusions

FHI and RP appear to share an unknown pathogenic pathway which predispose individuals to chronic, low-grade inflammation that may be involved in the development of similar clinical pictures [6, 7]. It has not been studied whether patients with both coexisting diseases have the same incidence of these complications (cataracts, macular edema) or if the presence of both has an added risk in the development or progression of these common findings. It is of special attention that this patient had not developed either of them, though the initial appearance of FHI signs had only recently been discovered, and cataracts and ocular hypertension secondary to FHI are usually present later in the history of the disease.

Due to it’s rarity, inverse RP has not been studied as classic RP has and therefore histologic, cellular and molecular findings are thought to be somewhat similar in both variants [1, 2]. It has been theorized that cases of inverse RP are in fact cone-rod dystrophies with macular affectation in which cones show early involvement either before rods start to dysfunction or the loss of both subsets of photoreceptors occurs simultaneously [1, 2]. The slight difference between them is the tendency of inverse RP to remain in the posterior pole and respect peripheral visual fields whereas CRD usually progresses until the whole visual field is affected.

 In any of both scenarios, FHI has not been previously reported as a coexisting disease. The thought that the inflammatory response triggered in the degenerative process leading to photoreceptor death in retinal dystrophies may be somewhat related to the unknown immunogenic pathways activated in patients with FHI gives hope that, if identified, it may become a target in the prevention and treatment of inflammatory complications in both diseases. This association also suggests FHI may be a triggered response genetically predisposed by a yet unidentified factor that may be related to the mutations responsible for RP. Our aim is to report FHI in a different variant of RP so that it becomes a disease specifically searched for in all subclasses of RP.

## Abbreviations

RP: retinitis pigmentosa
FHI: Fuchs heterochromic iridocyclitis
VA: visual acuity
BCVA: best corrected visual acuity
OD: right eye
OS: left eye
RPE: retinal pigment epithelium
CRD: cone-rod dystrophy
